# Pathway-specific metabolome analysis with ^18^O_2_-labeled *Medicago truncatula* via a mass spectrometry-based approach

**DOI:** 10.1007/s11306-018-1364-6

**Published:** 2018-05-11

**Authors:** Kota Kera, Dennis D. Fine, Daniel J. Wherritt, Yoshiki Nagashima, Norimoto Shimada, Takeshi Ara, Yoshiyuki Ogata, Lloyd W. Sumner, Hideyuki Suzuki

**Affiliations:** 1 Department of Research and Development, Kazusa DNA Research Institute, Kisarazu, Chiba 292-0818 Japan; 2 Plant Biology Division, The Samuel Roberts Noble Foundation, 2510 Sam Noble Parkway, Ardmore, OK USA; 3 Thermo Fisher Scientific, Yokohama, 221-0022 Japan; 4 Graduate School of Life and Environmental Sciences, Osaka Prefecture University, Osaka, 599-8531 Japan; 5 Present Address: Graduate School of Engineering, Tohoku University, Sendai, Miyagi 980-8579 Japan; 6 Present Address: Department of Biochemistry, Bond Life Science Center, University of Missouri, 1201 Rollins, Columbia, MO 65211 USA; 7 Present Address: Department of Chemistry, University of Texas at San Antonio, One UTSA Circle, San Antonio, TX 78249 USA; 8 Present Address: TOKIWA Phytochemical Co., Ltd., Sakura, Chiba 285-0801 Japan; 9 Present Address: Graduate School of Agriculture, Kyoto University, Kyoto, 611-0011 Japan

**Keywords:** Stable-isotope, Untargeted metabolome analysis, Flavonoid, *Medicago truncatula*, Metabolite modification

## Abstract

**Introduction:**

Oxygen from carbon dioxide, water or molecular oxygen, depending on the responsible enzyme, can lead to a large variety of metabolites through chemical modification.

**Objectives:**

Pathway-specific labeling using isotopic molecular oxygen (^18^O_2_) makes it possible to determine the origin of oxygen atoms in metabolites and the presence of biosynthetic enzymes (e.g., oxygenases). In this study, we established the basis of ^18^O_2_-metabolome analysis.

**Methods:**

^18^O_2_ labeled whole *Medicago truncatula* seedlings were prepared using ^18^O_2_-air and an economical sealed-glass bottle system. Metabolites were analyzed using high-accuracy and high-resolution mass spectrometry. Identification of the metabolite was confirmed by NMR following UHPLC–solid-phase extraction (SPE).

**Results:**

A total of 511 peaks labeled by ^18^O_2_ from shoot and 343 peaks from root were annotated by untargeted metabolome analysis. Additionally, we identified a new flavonoid, apigenin 4′-*O*-[2′-*O*-coumaroyl-glucuronopyranosyl-(1–2)-*O*-glucuronopyranoside], that was labeled by ^18^O_2_. To the best of our knowledge, this is the first report of apigenin 4′-glucuronide in *M. truncatula*. Using MS^n^ analysis, we estimated that ^18^O atoms were specifically incorporated in apigenin, the coumaroyl group, and glucuronic acid. For apigenin, an ^18^O atom was incorporated in the 4′-hydroxy group. Thus, non-specific incorporation of an ^18^O atom by recycling during one month of labeling is unlikely compared with the more specific oxygenase-catalyzing reaction.

**Conclusion:**

Our finding indicated that ^18^O_2_ labeling was effective not only for the mining of unknown metabolites which were biosynthesized by oxygenase-related pathway but also for the identification of metabolites whose oxygen atoms were derived from oxygenase activity.

**Electronic supplementary material:**

The online version of this article (10.1007/s11306-018-1364-6) contains supplementary material, which is available to authorized users.

## Introduction

It is well known that plants biosynthesize a wide variety of metabolites. A statistical approach has estimated that there are over 1,060,000 metabolites in plant species (Afendi et al. 2012). Although these are often beneficial in both plant physiology and for drug development (Newman and Cragg 2012). only a small proportion of these metabolites (50,897 metabolites were recorded in KNApSAcK) have been identified (Nakamura et al. 2014). Therefore, it is highly probable that metabolome analyses will lead to the identification of the unknown metabolites, uncover unknown biological phenomena and lead to advancements in drug development.

For large-scale and high-throughput metabolite analyses, chromatography-coupled to mass spectrometry (MS) is a very powerful tool due to its ability to detect a wide range of metabolites (Nakabayashi and Saito 2013). However, MS merely provides information on the mass and intensity of a large number of metabolite-derived peaks for metabolite, and ‘metabolite annotation’ is a crucial aspect of MS-based metabolomics. Annotation is typically performed by comparing tandem mass spectrometry (MS/MS) spectra of unknowns with spectra of known metabolites (Sumner et al. 2007). However, this approach cannot be applied to many rare plant-specific metabolites, because corresponding authentic standards are not available in many cases. Moreover, unknown metabolites are often annotated as ‘unknown’, and the information is not effectively used for further estimation (Iijima et al. 2008). Currently, advanced MS instrumentation with high accuracy and resolution enables a standard compound-independent annotation strategy in which the elemental composition is estimated based on accurate mass values (Makarov and Scigelova 2010; Ohta et al. 2010). To avoid incorrect assignment of elemental compositions, the natural average abundance of stable isotopes has been used (Kind and Fiehn 2006, 2007).Using a different approach, Giavalisco et al. prepared *Arabidopsis thaliana* labeled with ^13^C, ^15^N, and ^34^S, and estimated the elemental composition using the number of stable isotopes incorporated in the compound as an index (Giavalisco et al. 2011). Therefore, use of stable isotopes is effective for the estimation of elemental composition.

^13^C, ^15^N, and ^34^S have also been used as a tag or tracer, and are sometimes enriched by feeding plants with ^13^CO_2_, ^15^N-, or ^34^S-inorganic salts during cultivation (Glaser et al. 2014; Harada et al. 2006; Huege et al. 2007; Nakabayashi et al. 2013). In addition, ^18^O also has potential roles in metabolite flux analysis and gene mining. In higher plants, oxygen atoms are incorporated into compounds from carbon dioxide, water, or molecular oxygen by each responsible enzyme. Among them, oxygenases (EC1.13 and EC1.14) are well known for transferring an oxygen atom from molecular oxygen, and not from carbon dioxide or water, into metabolites (Hayaishi et al. 1955; Mason et al. 1955). In 2004, Hecht et al. labeled hop (*Humulus lupulus*) with ^18^O_2_ (molecular oxygen) in vivo and analyzed the origin of the oxygen atoms of humulone and cohumulone by nuclear magnetic resonance (NMR) spectrometry (Hecht et al. 2004). The result indicated that oxygen atoms from ^18^O_2_ were distinguishable from unlabeled O_2_. Further, in vivo labeling with ^18^O_2_ can be effective for suggesting the existence of an oxygenase in a biosynthetic pathway. However, there are no reports that have applied ^18^O_2_ (molecular oxygen) for MS-based comprehensive metabolome analysis.

## Materials and methods

### Plant materials

The unlabeled and labeled *Medicago truncatula*, Jemalong A17, were prepared as described previously (Kera et al. 2014). Seeds were sterilized with 70% ethanol and 1% (v/v) sodium hypochlorite solution, and rinsed with sterile water. Four seeds were sown on an agar medium, prepared in a 500 mL sealed glass bottle, and were grown for 5 weeks under the following conditions: temperature, 25 °C and a day/night rhythm of 18 h/6 h. The medium contained 1 mM potassium phosphate monobasic, 30 µM boric acid, 11 µM manganese(II) chloride, 1 µM/zinc chloride, 1 mM potassium chloride, 1 mM molybdic acid (sodium salt), 3.2 µM cupric chloride, 84 nM cobalt chloride, 1 mM calcium chloride, 50 µM Fe-ethylenediaminetetraacetic acid (EDTA), 1 mM magnesium sulfate, 10 mM ammonium nitrate, 10 mM potassium nitrate, 0.5% glucose, 1× Gamborg’s Vitamin Solution (Sigma-Aldrich, USA), and 0.8% Bacto Agar (Becton Dickinson, USA). During cultivation, 1 L of ^18^O-labeled air was exchanged daily. The plants were carefully collected from the bottle and the roots were washed with sterile water to remove agar medium. The shoots and roots were then separated with a scalpel and immediately frozen using liquid nitrogen. The plants were labeled with ^13^C [^13^C_6_-glucose (ISOTEC, Korea) in medium and ^13^C-labeled air (388 ppm ^13^C–CO_2_ and balance air; Tatsuoka, Japan)], ^15^N [^15^N-ammonium nitrate (SI Science, Japan) and 10 mM ^15^N-potassium nitrate (SI Science, Japan)], ^18^O [^18^O-labeled air (21.7% ^18^O–O_2_, 0.0394% CO_2_, and balance N_2_; Tatsuoka, Japan)], and ^34^S [^34^S-magnesium sulfate (SI Science, Japan)]. Details of the sample preparation methods are also available in the database Metabolonote (http://metabolonote.kazusa.or.jp/SE37:/).

### Analysis of the labeling efficiencies for ^13^C and ^15^N by sealed combustion method

The whole plants were lyophilized and ground by mortal. Samples were placed in quartz tubes that contained copper oxide. In the presence of Cu and Ag foil, the tubes were evacuated, sealed, and heated at 500 °C for 30 min and at 850 °C for 2 h, followed by cooling to room temperature. After combustion, the sample gas purified by cold trapping technique was analyzed by mass spectrometry RMI-2 (Hitachi, Japan).

### Liquid chromatography–mass spectrometry (LC–MS) measurement

The analysis was performed as described previously (Kera et al. 2014). The metabolites, extracted with 80% methanol (Optima LC/MS; Thermo Fisher Scientific, USA) from approximately 0.25 mg of the lyophilized plants, were injected in a liquid chromatography (LC)–Orbitrap–MS [LC, Agilent 1100 series (Agilent, USA); LTQ–Orbitrap (Thermo Fisher Scientific, USA) or LC, Dionex Ultimate 3000 (Thermo Fisher Scientific, USA); Orbitrap Fusion (Thermo Fisher Scientific, USA)]. The metabolites were separated using a C_18_ reversed-phase column (TSKgel ODS-100V; 4.6 × 250 mm, 5 µm; TOSOH, Japan) with a flow rate of 500 µL min^−1^ and the following gradient: water + 0.1% formic acid/acetonitrile + 0.1% formic acid, 3/97 to 97/3% (0.0–45.0 min), 3/97% (45.1–50.0 min), and 3/97% (50.1–57.0 min). Full mass scans were recorded with high resolution (LTQ–Orbitrap, 60,000; Orbitrap Fusion, 120,000) and MS/MS were acquired on the five most intense ions from each full scan by ion trap or Orbitrap Fusion, covering a mass range from *m*/*z* 100.0–1500.0.

### Data analysis

Data analysis was performed as described previously (Kera et al. 2014). All raw data files were converted into text files using MSGet (http://www.kazusa.or.jp/komics/software/MSGet), and peaks were extracted by PowerFT (http://www.kazusa.or.jp/komics/software/PowerGet/) (Sakurai et al. 2014). Unlabeled and blank data were aligned using PowerMatch (http://www.kazusa.or.jp/komics/software/PowerGet/) with manual curation, and valid peaks were selected after background correction. In the annotation process, the primary database search and the primary estimation of elemental composition were performed using PowerMatch and MFSearcher within a mass accuracy of 3 ppm (http://webs2.kazusa.or.jp/mfsearcher/) (Sakurai et al. 2013). Labeling data were further analysis by ShiftedIonsFinder (Kera et al. 2014).

To find the isotopic peaks in the labeled data and associate them with monoisotopic peaks from the unlabeled data, the labeled peak search was performed with the parameters as follows: Max fold, C = 100, N = 50, O = 50, S = 50; Mass difference = 3 ppm; and RT difference = 1. In flavonoid-like peak analysis, candidate peaks with chemical modification, such as glycosylation and acylation, were searched by comparing the peak list containing mass information of aglycone of typical flavonoids against the peak list from an unlabeled sample using ShiftedIonsFinder. Xylosylation (Xyl) (C_5_H_8_O_4_, *m*/*z* 132.04226), glucosylation (Glc) (C_6_H_10_O_5_, *m*/*z* 162.05282), rhamnosylation (Rha) (C_6_H_10_O_4_, *m*/*z* 146.05791), glucuronidation (GlcUA) (C_6_H_8_O_6_, *m*/*z* 176.03209), cinnamoylation (Cinnamoyl) (C_9_H_6_O_1_, *m*/*z* 130.04186), coumaroylation (Coumaroyl) (C_9_H_6_O_2_, *m*/*z* 146.03678), caffeoylation (Caffeoyl) (C_9_H_6_O_3_, *m*/*z* 162.03169), feruloylation (Feruloyl) (C_10_H_8_O_3_, *m*/*z* 176.04734), malonylation (Malonyl) (C_3_H_2_O_3_, *m*/*z* 89.0003939), and succinylation, (Succinyl) (C_4_H_4_O_3_, *m*/*z* 100.016044) were selected as modification groups. The settings were as follows: Max fold, Glc = 3, Rha = 3, GlcUA = 3, Cinnamoyl = 3, Coumaroyl = 3, Caffeoyl = 3, Feruloyl = 3, Malonyl = 3, Succinyl = 3; Mass difference = 3 ppm; RT difference = 60. The exported file from ShiftedIonsFinder was arranged by Excel.

### UHPLC–ESI–QTOF–MS/MS measurement

The metabolites, extracted with 80% methanol were injected in a Waters ACQUITY UPLC (Waters, USA) coupled to a Bruker maXis Impact ESI–QTOF–MS system having a mass resolution of ∼ 40,000 (Bruker Daltonics, USA). The identification of the metabolite was confirmed by NMR following UHPLC–solid-phase extraction (SPE) using a Bruker/Spark Holland Prospekt II SPE system as described previously (Qiu et al. 2016). Details were provided in supplemental data 4.

## Results and discussion

### Establishment of an economical sealed-glass bottle system

**Fig. 1:**
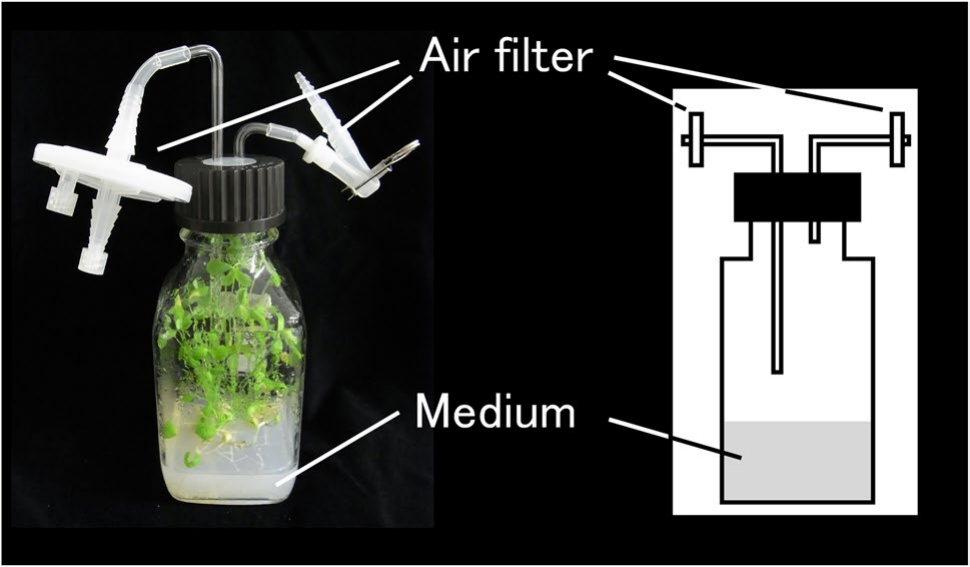
The sealed-glass bottle system for economical labeling. The normal or labeled air was blown into the bottle through an air filter. After gas exchange, the flow channels of the filter were closed. The other labeled nutrients were supplied in the medium beforehand

Although many stable isotopes, such as, ^13^C, ^15^N, and ^34^S, have been used for in vivo labeling, ^18^O (especially in its molecular oxygen form) has not been effectively used. A reported ^13^CO_2_ labeling procedure (Huege et al. 2007) is not suited for ^18^O_2_ labeling, because ^18^O-labeled air is too expensive to fill most growth chambers. Hecht et al. previously proposed an economical ^18^O_2_ labeling system to produce ^18^O-labeled hop (Hecht et al. 2004). In their system, several cones were enclosed in glass vessels filled with 20% ^18^O_2_ and 80% argon, and were incubated for 14 days without gas exchange. Therefore, we used a sealed-glass bottle with air filters (ADVANTEC, Japan) (Fig. 1) for gas exchange. Additionally, unlabeled and ^13^C-, ^15^N-, and ^34^S-labeled samples were prepared using the same setup, respectively. The labeling efficiencies for ^13^C-labeled and ^15^N-labeled samples were 59.6 and 78.8%, respectively, while those of unlabeled sample were 1.09 and 0.365%, respectively. These were not as high compared with those in previous reports, in which greater than 90% efficiency was achieved using a growth chamber (Giavalisco et al. 2011). In order to grow each labeling plants in equal condition, ^13^C-labeled air was exchanged daily in this study. However, continuous gas flow might improve it. For ^15^N-labeling, exchanging medium during long cultivation might effective. To enable exchange of medium, we plan to use liquid medium and modify Leonard jar (Leonard 1943) to keep sealed and exchange the gas in the future. Moreover, 78.8% of ^15^N labeling efficiency was not perfect but it seemed to be enough for further curation of the elemental composition. Because incorporation of ^18^O or ^34^S cannot be measured directly, the 80% methanol extraction of unlabeled, ^18^O-labeled, and ^34^S-labeled shoots were analyzed by LC–Orbitrap–MS using a mass accuracy of 3 ppm. The incorporation was manually confirmed in the mass spectra using Xcalibur (Thermo Fisher Scientific, USA) (data not shown). These results suggest that this labeling system works, although that there is still room for improvement in labeling efficiency.

### Untargeted metabolite annotation of peaks able to be labeled by ^18^O_2_

For target analysis, whether a target metabolite is labeled by ^18^O_2_ or not, was investigated by searching for the calculated *m*/*z* values of ^18^O-labeled peaks in the ^18^O_2_ labeled sample. Here, this is calculated by adding the *m*/*z* difference between ^16^O and ^18^O of 2.0042462 to that of a target monoisotopic peak. However, this approach is not applicable to non-target analyses, because the monoisotopic *m*/*z* values of peaks that can be labeled by ^18^O_2_ are not assigned. One effective approach is to compare mass spectra from an unlabeled sample with those from a ^18^O_2_ labeled sample, and associate the peaks in the unlabeled sample with the peaks in the ^18^O_2_ labeled sample based on the specified *m*/*z* difference responsible for ^18^O-labeling. Therefore, the comprehensive search for peaks labeled by ^18^O_2_ can be accomplished in four steps: (1) preparation of an unlabeled peak list from unlabeled samples with tight intensity filter to reduce noise: (2) preparation of an ^18^O-labeled peak list from ^18^O_2_ labeled samples with a loose intensity filter to pick tiny peaks; (3) search for peaks labeled by ^18^O_2_ by association of the unlabeled peak list with the ^18^O-labeled peak list using ShiftedIonsFinder (Kera et al. 2014), and (4) curation of the search results by manually checking the mass spectra. Initially, a total of 3,417 peaks and 3,518 peaks were extracted as unlabeled peaks from *M. truncatula* shoots and roots, respectively. Because analysis using ShiftedIonsFinder requires manual curation of the mass spectra, we selected peaks labeled by ^18^O_2_ according to the search result of ShiftedIonsFinder and the existence of MS^2^ information for further annotation steps. Finally, a total of 511 peaks and 353 peaks were extracted as peaks labeled by ^18^O_2_ from *M. truncatula* shoots and roots, respectively (Supplement data 1, 2).

The elemental compositions of peaks labeled by ^18^O_2_ were first predicted using MFSearcher (Sakurai et al. 2013). Because multiple elemental compositions are generally predicted for peaks with high *m*/*z* values, even if the deviation of *m*/*z* value is within 1 ppm, the predicted elemental compositions were manually curated according to a strategy in which the number of stable isotopes incorporated in the compound is used as an index (Giavalisco et al. 2011; Kera et al. 2014). As well as detecting ^18^O-labeled peaks, the corresponding ^13^C-, ^15^N-, and ^34^S-labeled peaks labeled by ^18^O_2_ were found from each labeled sample by comparing with unlabeled peak list using ShiftedIonsFinder. Considering that the labeling efficiency was not enough to determine the max number of each element, we excluded the elemental compositions in which the number of each element was less than that of detected. As a result, the single elemental composition increased from about 4% (shoot: 20 peaks, 3.9%; root: 14 peaks, 4.0%) to about 60% (shoot: 311 peaks, 60.9%; root: 222 peaks, 62.9%), while multiple elemental compositions decreased from about 95% (shoot: 489 peaks, 95.7%; root: 335 peaks, 94.9%) to about 38% (shoot: 193 peaks, 37.8%; root: 121 peaks, 34.3%) (Table 1). Among peaks having a single elemental composition, 195 peaks from shoot and 161 peaks from root were present in databases (see “Materials and methods”), suggesting that many secondary metabolites can be labeled with ^18^O_2_ by oxygenases (Table 2).

**Table 1:** The status of estimating elemental composition within 3 ppm

Status of elemental composition	Shoot				Root			
	Automatic estimation		Manual curation		Automatic estimation		Manual curation	
	Number (peaks)	Rate (%)	Number (peaks)	Rate (%)	Number (peaks)	Rate (%)	Number (peaks)	Rate (%)
Single	20	3.9	311	60.9	14	4.0	222	62.9
Multiple	489	95.7	193	37.8	335	94.9	121	34.3
No hits	2	0.4	7	1.4	4	1.1	10	2.8
Total	511	100.0	511	100.0	353	100.0	353	100.0

**Table 2:** The category of database search results for peaks having a single elemental composition

Category	Shoot		Root	
	Number (peaks)	Rate (%)	Number (peaks)	Rate (%)
Aminocarboxylic acid	13	6.7	3	1.9
Nucleoside	2	1.0	0	0.0
Organic acid	2	1.0	1	0.6
Peptide	1	0.5	2	1.2
Isoprenoid	66	33.8	27	16.8
Phenolic	31	15.9	89	55.3
Alkaloid	2	1.0	1	0.6
Other	28	14.4	18	11.2
Multiple	48	24.6	19	11.8
SEC (there are some DB hits, but unlikely)	2	1.0	1	0.6
Total	195	100.0	161	100.0

### Identification of ^18^O-labeled unknown flavonoids

**Fig. 2:**
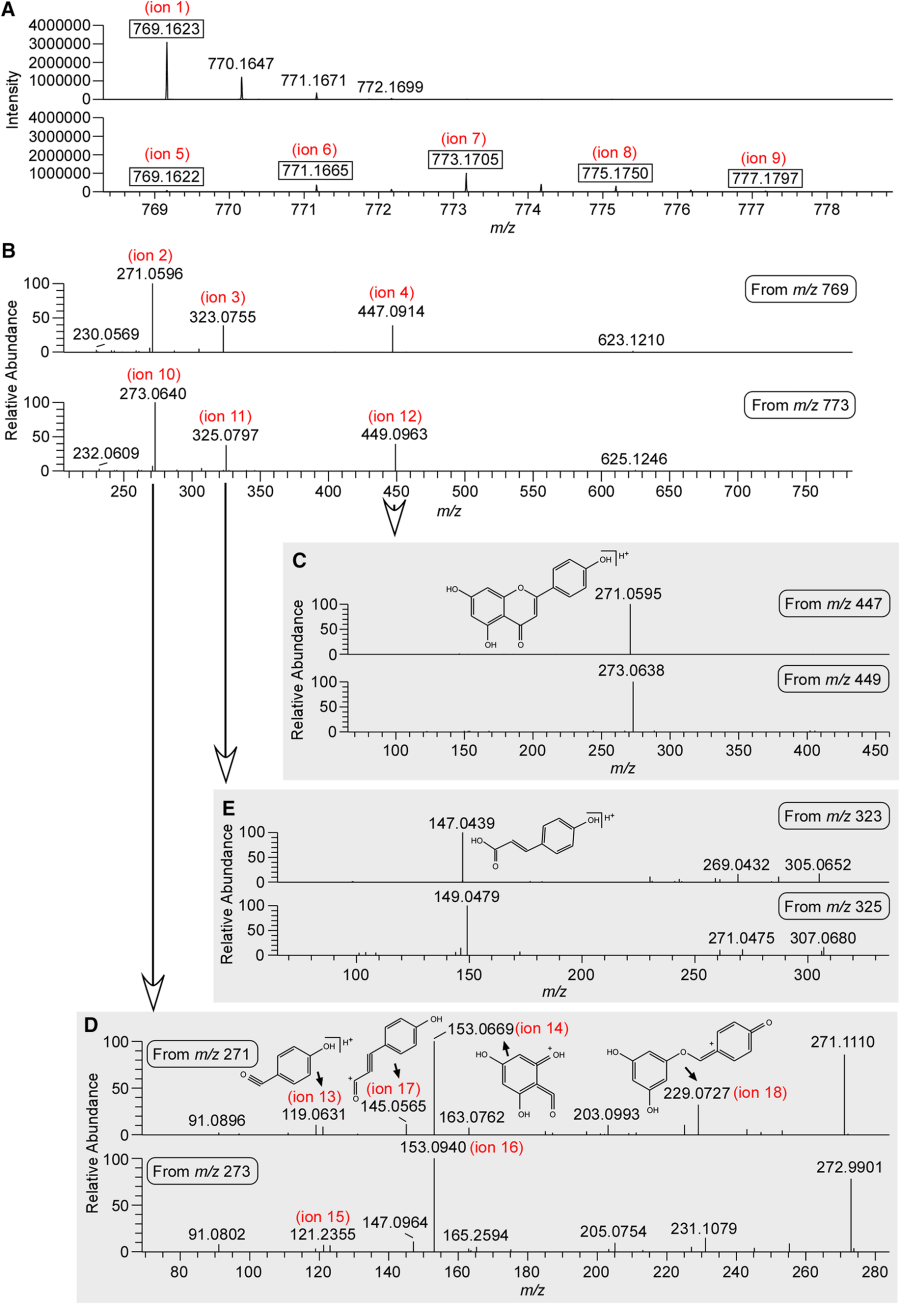
MS^n^ analysis of peak no. 312, apigenin 4′-*O*-[2′-*O*-coumaroyl-glucuronopyranosyl-(1–2)-*O*-glucuronopyranoside], by LC–orbitrap-fusion. **a** The full scan of unlabeled (above) and ^18^O-labeled sample (bottom) by Orbitrap. **b** MS^2^ analysis of peak no. 312 (above) and ion 7 (bottom) by Orbitrap. **c** MS^3^ analysis of ion 4 (above) and ion 12 (bottom) by Orbitrap. **d** MS^3^ analysis of ion 2 (above) and ion 10 (bottom) by ion trap. **e** MS^3^ analysis of ion 3 (above) and ion 11 (bottom) by Orbitrap

**Fig. 3:**
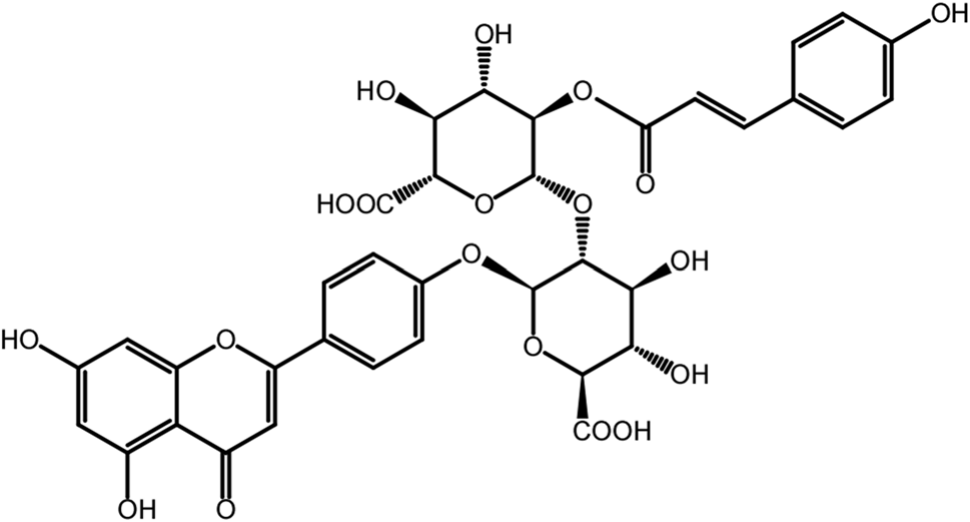
The structure of apigenin 4′-*O*-[2′-*O*-coumaroyl-glucuronopyranosyl-(1–2)-*O*-glucuronopyranoside]. It was identified using a UHPLC–MS–SPE–NMR system and the detail information was shown in supplemental data 4

As described in a previous report, ShiftedIonsFinder is useful for finding candidates of known and unknown flavonoids by calculating theoretical *m*/*z* values by adding modification groups to aglycone like a building block (Kera et al. 2014). Potential flavonoids were searched for within the 511 peaks of *M. truncatula* shoot with regard to some modification groups. As a result, 26 peaks were picked up as flavonoid candidates (Supplement data 3). As an example, we focused on peak No. 312 (ion 1: *m*/*z* 769.1623 in Fig. 2a), one of the flavonoid candidates whose mass was not matched to referenced databases. We first attempted to estimate its structure by a MS-based approach. This peak yielded three candidate elemental compositions, C_47_H_29_O_9_P_1_, C_36_H_32_O_19_, and C_54_H_24_O_6_ within 3 ppm, and 38 candidate flavonoids. In order to estimate its detailed structure, additional MS^n^ analyses with high resolution and high mass accuracy using Orbitrap Fusion (Thermo Fisher Scientific, USA) were performed. At first, three major fragments (ion 2: *m*/*z* 271.0596; ion 3: *m*/*z* 323.0756; and ion 4: *m*/*z* 447.0916) were detected by MS^2^ analysis from the parent monoisotopic ion (Fig. 2b). In positive electrospray ionization-mode fragmentation of flavonoid glycosides, it is well known that after cleavage of glycosidic bonds between the glycoside group and aglycone (Cuyckens and Claeys 2004), the product ion for a flavonoid aglycone results. It is strongly suggested that ion 2 at *m*/*z* 271.0596 is the aglycone. Among the 38 candidate flavonoids suggested by ShiftedIonsFinder, the structure having such an aglycone was [Aglycone + 2 GlcUA + Coumaroyl + H]^+^ (Table 3). This estimation indicates that ion 3 (*m*/*z* 323.0756) was [GlcUA + Coumaroyl + H]^+^, and ion 4 (*m*/*z* 447.0916) was [Aglycone + GlcUA + Coumaroyl + H]^+^. In fact, MS^3^ analysis of ion 3 and ion 4 supported the estimation, and the fragmentation pattern of ion 2 agreed with that of apigenin using *m*/*z*Cloud (https://www.m/zcloud.org/; Thermo Fisher Scientific, USA). Taken together, this unknown peak No. 312 (*m*/*z* 769.1624) was annotated as [Apigenin] + 2 GlcUA + Coumaroyl + [H]^+^, and the elemental composition was C_36_H_32_O_19_. The structure was finally identified as apigenin 4′-*O*-[2′-*O*-coumaroyl-glucuronopyranosyl-(1–2)-*O*-glucuronopyranoside] using a UHPLC–MS–SPE–NMR system (Fig. 3, Supplement data 4) (Sumner et al. 2014, 2015). To date, many types of apigenin glucuronides have been reported from *M. truncatula* (Kowalska et al. 2007; Marczak et al. 2010) and *Medicago sativa* (Stochmal et al. 2001a, b). Apigenin 7-*O*-[2′-*O*-coumaroyl-glucuronopyranosyl-(1–2)-*O*-glucuronopyranoside] has already been reported (Marczak et al. 2010). Interestingly, glucuronidation at the 4′ position of apigenin was found in *M. sativa*, but not in *M. truncatula*. Additionally, we also identified peak No. 335 (*m*/*z* 799.1730) as apigenin 4′-*O*-[2′-*O*-feruloyl-glucuronopyranosyl-(1–2)-*O*-glucuronopyranoside], which was found in *M. sativa*. This result is the first report of apigenin 4′-glucuronides present in *M. truncatula*. There were other peaks with the same mass values (*m*/*z* 769.1624 and *m*/*z* 799.1730) at different retention times in our data, suggesting the presence of structural isomers in *M. truncatula*.

**Table 3:** Thirty-eight structural candidates of peak No. 312 (*m*/*z* 769.1624) by ShiftedIonsFinder

Ave. mass (detected)	Ave. mass (actual)	Name	Class	Ioniz.Mode	Xyl (C_5_H_8_O_4_): lag (times)	Rha (C_6_H_10_O_4_): lag (times)	Glc (C_6_H_10_O_5_): lag (times)	GlcUA (C_6_H_8_O_6_): lag (times)	Cinnamoyl (C_9_H_6_O_1_): lag (times)	Coumaroyl (C_9_H_6_O_2_) : lag (times)	Caffeoyl (C_9_H_6_O_3_): lag (times)	Feruloyl (C_10_H_8_O_3_): lag (times)	Malonyl (C_3_H_2_O_3_): lag (times)	Succinyl (C_4_H_4_O_3_): lag (times)
255.0652	254.0579	Chrysin (C_15_H_10_O_4_)	Flavone	[M + H]^+^	0	0	0	2	0	0	1	0	0	0
255.0652	254.0579	Daidzein (C_15_H_10_O_4_)	Isoflavone	[M + H]^+^	0	0	0	2	0	0	1	0	0	0
271.0601	270.0528	Sulphuretin (C_15_H_10_O_5_)	Aurone	[M + H]^+^	0	0	0	2	0	1	0	0	0	0
271.0601	270.0528	Apigenin (C_15_H_10_O_5_)	Flavone	[M + H]^+^	0	0	0	2	0	1	0	0	0	0
271.0601	270.0528	Genistein (C_15_H_10_O_5_)	Isoflavone	[M + H]^+^	0	0	0	2	0	1	0	0	0	0
271.0601	271.0606	Pelargonidin (C_15_H_11_O_5_)	Anthocyanidin	[M]^+^	0	0	0	2	0	1	0	0	0	0
273.0757	272.0685	"Naringenin chalcone, butein (C_15_H_12_O_5_)"	Chalcone	[M + H]^+^	0	0	1	0	0	0	1	0	2	0
273.0757	272.0685	Naringenin (C_15_H_12_O_5_)	Flavanone	[M + H]^+^	0	0	1	0	0	0	1	0	2	0
275.0914	274.0841	Phloretin (C_15_H_14_O_5_)	Dihydrochalcone	[M + H]^+^	0	0	0	1	0	1	0	0	2	0
287.0550	286.0477	Aureusidin (C_15_H_10_O_6_)	Aurone	[M + H]^+^	0	0	0	2	1	0	0	0	0	0
287.0550	286.0477	Luteolin (C_15_H_10_O_6_)	Flavone	[M + H]^+^	0	0	0	2	1	0	0	0	0	0
287.0550	286.0477	Kaempferol (C_15_H_10_O_6_)	Flavonol	[M + H]^+^	0	0	0	2	1	0	0	0	0	0
287.0550	287.0556	Cyanidin (C_15_H_11_O_6_)	Anthocyanidin	[M]^+^	0	0	0	2	1	0	0	0	0	0
289.0707	288.0634	Pentahydroxychalcone (C_15_H_12_O_6_)	Chalcone	[M + H]^+^	0	0	1	0	0	1	0	0	2	0
289.0707	288.0634	Pentahydroxychalcone (C_15_H_12_O_6_)	Chalcone	[M + H]^+^	0	1	0	0	0	0	1	0	2	0
289.0707	288.0634	Pentahydroxychalcone (C_15_H_12_O_6_)	Chalcone	[M + H]^+^	1	0	0	0	0	0	0	1	2	0
289.0707	288.0634	Pentahydroxychalcone (C_15_H_12_O_6_)	Chalcone	[M + H]^+^	1	0	0	0	0	0	1	0	1	1
289.0707	288.0634	Eriodictyol (C_15_H_12_O_6_)	Flavanone	[M + H]^+^	0	0	1	0	0	1	0	0	2	0
289.0707	288.0634	Eriodictyol (C_15_H_12_O_6_)	Flavanone	[M + H]^+^	0	1	0	0	0	0	1	0	2	0
289.0707	288.0634	Eriodictyol (C_15_H_12_O_6_)	Flavanone	[M + H]^+^	1	0	0	0	0	0	0	1	2	0
289.0707	288.0634	Eriodictyol (C_15_H_12_O_6_)	Flavanone	[M + H]^+^	1	0	0	0	0	0	1	0	1	1
289.0707	288.0634	Dihydrokaempferol (C_15_H_12_O_6_)	Flavanonol	[M + H]^+^	0	0	1	0	0	1	0	0	2	0
289.0707	288.0634	Dihydrokaempferol (C_15_H_12_O_6_)	Flavanonol	[M + H]^+^	0	1	0	0	0	0	1	0	2	0
289.0707	288.0634	Dihydrokaempferol (C_15_H_12_O_6_)	Flavanonol	[M + H]^+^	1	0	0	0	0	0	0	1	2	0
289.0707	288.0634	Dihydrokaempferol (C_15_H_12_O_6_)	Flavanonol	[M + H]^+^	1	0	0	0	0	0	1	0	1	1
291.0863	290.0790	Leucopelargonidin (C_15_H_14_O_6_)	Leucoanthocyanidin	[M + H]^+^	0	0	0	1	1	0	0	0	2	0
291.0863	290.0790	Catechin (C_15_H_14_O_6_)	Flavanol	[M + H]^+^	0	0	0	1	1	0	0	0	2	0
303.0863	302.0790	Hesperetin (C_16_H_14_O_6_)	Flavanone	[M + H]^+^	1	0	0	0	0	0	1	0	2	0
305.0656	304.0583	Dihydroquercetin (C_15_H_12_O_7_)	Flavanonol	[M + H]^+^	0	0	1	0	1	0	0	0	2	0
305.0656	304.0583	Dihydroquercetin (C_15_H_12_O_7_)	Flavanonol	[M + H]^+^	0	1	0	0	0	1	0	0	2	0
305.0656	304.0583	Dihydroquercetin (C_15_H_12_O_7_)	Flavanonol	[M + H]^+^	1	0	0	0	0	1	0	0	1	1
307.0812	306.0740	Leucocyanidin (C_15_H_14_O_7_)	Leucoanthocyanidin	[M + H]^+^	0	0	0	0	0	0	0	1	1	2
307.0812	306.0740	Leucocyanidin (C_15_H_14_O_7_)	Leucoanthocyanidin	[M + H]^+^	0	0	0	0	0	0	1	0	0	3
307.0812	306.0740	Gallocatechin (C_15_H_14_O_7_)	Flavanol	[M + H]^+^	0	0	0	0	0	0	0	1	1	2
307.0812	306.0740	Gallocatechin (C_15_H_14_O_7_)	Flavanol	[M + H]^+^	0	0	0	0	0	0	1	0	0	3
321.0605	320.0532	Dihydromyricetin (C_15_H_12_O_8_)	Flavanonol	[M + H]^+^	0	1	0	0	1	0	0	0	2	0
321.0605	320.0532	Dihydromyricetin (C_15_H_12_O_8_)	Flavanonol	[M + H]^+^	1	0	0	0	1	0	0	0	1	1
323.0761	322.0689	Leucodelphinidin (C_15_H_14_O_8_)	Leucoanthocyanidin	[M + H]^+^	0	0	0	0	0	1	0	0	0	3

Lag (times) means the number of each possible modification group (Kera et al. 2014)

### Discussion of ^18^O-labeled positions in the metabolite

An important aim of this study is to estimate the ^18^O-labeled position in metabolites with MS-based information. For peak No. 312 (*m*/*z* 769.1624, C_36_H_32_O_19_, apigenin 4′-*O*-[2′-*O*-coumaroyl-glucuronopyranosyl-(1–2)-*O*-glucuronopyranoside]), five corresponding ions (ion 5: *m*/*z* 769.1622; ion 6: *m*/*z* 771.1665; ion 7: *m*/*z* 773.1705; ion 8: *m*/*z* 775.1750; and ion 9: *m*/*z* 777.1797) were detected, and the intensity of ion 7 was highest in the ^18^O-labeled sample (Fig. 2a). Although we have not investigate whether the ^18^O abundance ratio changed during cultivation, that of peak No. 312 from several five weeks old plants was similar. This indicates that the labeled molecule containing two ^18^O atoms was more abundant, and also that there are two major oxygenase-catalyzed steps in the biosynthetic pathway of apigenin 4′-*O*-[2′-*O*-coumaroyl-glucuronopyranosyl-(1–2)-*O*-glucuronopyranoside]. In order to estimate the ^18^O-labeled position, MS^n^ analysis for the monoisotopic ion and ion 7: *m*/*z* 773.1705 was performed using Orbitrap Fusion. In MS^2^ analysis of ion 7: *m*/*z* 773.1705, the fragmentation pattern was similar to that of the monoisotopic ion, containing three major fragments (ion 10: *m*/*z* 273.0640; ion 11: *m*/*z* 325.0797; and ion 12: *m*/*z* 449.0963) (Fig. 2b). Comparison with the mass of each responsible ion indicated that apigenin had one ^18^O atom, [GlcUA + Coumaroyl] (2′-*O*-coumaroyl-glucuronopyranoside) had one ^18^O atom, and [Apigenin + GlcUA] (apigenin 4′-*O*-glucuronopyranoside) had one ^18^O atom, respectively. Here, one of the two ^18^O atoms was thought to be incorporated in apigenin. Detection of the major fragment of *m*/*z* 273.0638 from ion 12: *m*/*z* 449.0963 in MS^3^ analysis supports this estimation (Fig. 2c). In some, but not all, cases, MS^n^ analysis can indicate a more confined ^18^O-labeled position. In MS^3^ analysis of apigenin (ion 2), two characteristic fragments (ion 13: *m*/*z* 119.0631 and ion 14: *m*/*z* 153.0669) are known retro-Diels–Alder fragment ions (Fig. 2d). Focusing on the responsible ions (ion 15: *m*/*z* 121.2355 and ion 16: *m*/*z* 153.0940) in MS^3^ analysis of ion 10, the ^18^O responsible mass shift was detected between ion 13 and ion 15, but not between ion 14 and ion 16 (Fig. 2d). In addition to ion 13, other fragment ions containing a 4′-hydroxy group (ion 17: *m*/*z* 145.0565 and ion 18: *m*/*z* 229.0727) also showed the ^18^O mass shift, whereas ion 16 contains all four oxygen atoms except the 4′-hydroxy group. Hence, the ^18^O atom was specifically incorporated into the 4′-hydroxy group of apigenin. According to the apigenin biosynthetic pathway, the hydroxy groups at positions 1, 4, 5, and 7 are derived from malonyl CoA, which is synthesized by an oxygenase-independent pathway, and the 4′-hydroxy group is derived from *p*-coumaroyl CoA by cinnamic acid 4-hydroxylase (C4H), which is a cytochrome P450 monooxygenase (Saito et al. 2013). Thus, specific incorporation into the 4′-hydroxy group is reasonable and indicates that non-specific incorporation of an ^18^O atom by recycling during one month of labeling is unlikely compared with the oxygenase-catalyzing reaction. On the other hand, by MS^3^ analysis of ion 3: *m*/*z* 323.0756 and ion 11: *m*/*z* 325.0797, one major fragment (*m*/*z* 147.0439 [Coumaroyl]) was detected from ion 3, and one major fragment (*m*/*z* 149.0479) was detected from ion 11, indicating that one ^18^O atom in ion 11 was derived from [Coumaroyl] (Fig. 2e). We could not determine the detailed labeling position by MS^n^ analysis, but an ^18^O atom was also thought to be incorporated at the C-4 position of [Coumaroyl] by C4H. Finally, tiny ion 8: *m*/*z* 775.1750 and ion 9: *m*/*z* 777.1797 were responsible for the incorporation in [GlcUA] (data not shown). Since UDP-glucuronic acid is biosynthesized in plants through both a UDP-glucose 6-dehydrogenase-catalyzing reaction, in which H_2_O is used as an oxygen source, and a *myo*-inositol oxygenase-catalyzing reaction, in which O_2_ is used (Roberts 1971), these weak ions might reflect the biosynthetic pathway in *M. truncatula*. Consequently, these data well supported that ion 7 containing two ^18^O atoms from [Coumaroyl] was more abundant.

## Concluding remarks

We have studied pathway-specific metabolome analysis using ^18^O_2_-labeled *M. truncatula*. This economical ^18^O_2_ labeling system enables whole plant labeling for over 1 month with ^18^O_2_-air. From untargeted analysis data, we succeeded in the identification of an unknown ^18^O-labeled flavonoid, apigenin 4′-*O*-[2′-*O*-coumaroyl-glucuronopyranosyl-(1–2)-*O*-glucuronopyranoside]. By MS^n^ analysis of this flavonoid in unlabeled and ^18^O_2_-labeled samples, we estimated that ^18^O atoms were specifically incorporated in apigenin, the coumaroyl group, and glucuronic acid. For apigenin, an ^18^O atom was specifically incorporated in the 4′-hydroxy group. This approach enables the determination of not only the presence of oxygenase in the biosynthetic pathway but also that the identification of oxygen atoms in metabolites derived from oxygenases. Therefore, pathway-specific metabolome analysis can be a key for linking metabolite information to enzymatic information.

## Electronic supplementary material

Below is the link to the electronic supplementary material.

Supplementary material 1 (XLSX 2263 KB)

Supplementary material 2 (XLSX 2011 KB)

Supplementary material 3 (XLSX 67 KB)

Supplementary material 4 (PPTX 2078 KB)
